# Implementing medication adherence interventions in four Dutch living labs; context matters

**DOI:** 10.1186/s12913-023-10018-4

**Published:** 2023-09-26

**Authors:** Stijn Hogervorst, Marcia Vervloet, Ruby Janssen, Ellen Koster, Marcel C. Adriaanse, Charlotte L. Bekker, Bart J. F. van den Bemt, Marcel Bouvy, Eibert R. Heerdink, Jacqueline G. Hugtenburg, Menno van Woerkom, Hanneke Zwikker, Caroline van de Steeg-van Gompel, Liset van Dijk

**Affiliations:** 1 Department of Health Sciences, Faculty of Science, Vrije Universiteit, Amsterdam, The Netherlands; 2 Amsterdam Public Health Research Institute, Amsterdam UMC, Location VUMC, De Boelelaan 1117, 1081 HV Amsterdam, The Netherlands; 3 Department of Pharmaceutical Care, Nivel, Netherlands Institute for Health Services Research, Utrecht, The Netherlands; 4 Hogeschool Utrecht, Lectorate Innovations in Healthcare Processes in Pharmacology, Utrecht, The Netherlands; 5 Utrecht Institute of Pharmaceutical Sciences, Divison of Pharmacoepidemiology and Clinical Pharmacology, Utrecht University, Utrecht, the Netherlands; 6 Department of Pharmacy, Radboud Institute for Health Sciences, Radboud University Medical Centre, Nijmegen, the Netherlands; 7 Department of Pharmacy, Sint Maartenskliniek, Nijmegen, the Netherlands; 8 Department of Clinical Pharmacology and Pharmacy, Amsterdam UMC, Location VUMC, De Boelelaan 1117, 1081 HV Amsterdam, The Netherlands; 9 Dutch Institute for Rational Use of Medicine (IVM), Utrecht, the Netherlands; 10 SIR Institute for Pharmacy Practice and Policy, Leiden, The Netherlands; 11 Department of PharmacoTherapy, Epidemiology and Economics (PTEE), Faculty of Mathematics and Natural Sciences, Groningen Research Institute of Pharmacy, University of Groningen, Groningen, Netherlands

**Keywords:** Context, Living labs, Real-world setting, Medication adherence, Implementation, Consolidated framework for implementation research (CFIR), Pharmaceutical care, Innovation

## Abstract

**Background:**

Despite the abundant availability of effective medication adherence interventions, uptake of these interventions into routine care often lacks. Examples of effective medication adherence interventions include telephone counseling, consult preparation and the teach-back method. Assessing context is an important step in understanding implementation success of interventions, but context is often not reported or only moderately described. This study aims to describe context-specific characteristics in four living labs prior to the implementation of evidence-based interventions aiming to improve medication adherence.

**Methods:**

A qualitative study was conducted within four living labs using individual interviews (*n* = 12) and focus groups (*n* = 4) with project leaders and involved healthcare providers. The four living labs are multidisciplinary collaboratives that are early adopters of medication adherence interventions in the Dutch primary care system. Context is defined as the environment or setting in which the proposed change is to be implemented. Interview topics to assess context were formulated based on the ‘inner setting’ and ‘outer setting’ domains of the Consolidated Framework for Implementation Research (CFIR). Interviews were recorded and transcribed verbatim. Transcripts were deductively analyzed.

**Results:**

A total of 39 community pharmacists, pharmacy technicians, general practitioners and a home care employee participated in the (focus group) interviews. All four living labs proved to be pharmacy-driven and characterized by a high regard for innovation by staff members, a positive implementation climate, high levels of leadership engagement and high compatibility between the living labs and the interventions. Two living labs were larger in size and characterized by more formal communication. Two living labs were characterized by higher levels of cosmopolitanism which resulted in more adaptable interventions. Worries about external policy, most notably lack of reimbursement for sustainment and upscaling of the interventions, were shared among all living labs.

**Conclusions:**

Contextual characteristics of four living labs that are early adopters of medication adherence interventions provide detailed examples of a positive implementation setting. These can be used to inform dissemination of medication adherence interventions in settings less experienced in implementing medication adherence interventions.

**Supplementary Information:**

The online version contains supplementary material available at 10.1186/s12913-023-10018-4.

## Background

Evidence shows that 44 to 77% of chronic medication users across different disease groups, countries and settings are non-adherent to their treatment, which diminishes treatment effects and increases symptom severity, mortality, hospitalization, healthcare utilization and costs [1–6]. Non-adherence occurs when a patient’s medication use behavior does not correspond with the prescribed medication intake regimen agreed upon with their healthcare providers [7]. Effective interventions have been developed to improve medication adherence such as educational interventions and interventions to increase motivation among patients [8, 9]. Examples include telephone counseling, consult preparation or the teach-back method [10–14]. Medication adherence interventions are complex because they frequently involve continuous monitoring of non-adherent subpopulations, the need to tailor approaches based on diverse patient barriers, and ongoing collaboration among healthcare providers. [15]. They often do not show long term effectiveness, which means interventions need to be fully integrated into healthcare systems to be able to provide ongoing treatment of non-adherent patients [16].

However, in general only 14% of effective healthcare interventions are implemented into routine care and their implementation takes an average of 17 years [17, 18]. Although no exact data are known, given their complexity, this most likely also holds true for medication adherence interventions. Interventions are developed in randomized controlled trials (RCTs), which eliminates all real world conditions with strict inclusion and exclusion criteria in favor of measuring effectiveness [19]. RCTs are essential for developing and selecting suitable interventions, but a deep understanding of the real world context is crucial in order to integrate complex interventions into a real world healthcare setting [20]. That is because healthcare providers often lack the necessary resources such as time, money, organization or skills to implement existing interventions into their own practices or to scale-up existing implementation efforts. In order to help implement these effective medication adherence interventions into daily clinical practice, more knowledge on how they work is therefore needed in order to implement more effective medication adherence interventions into daily clinical practice.

To successfully implement a complex intervention into routine care, assessing context is an important step widely recognized in dissemination and implementation science [21–23]. It serves as the basis of all subsequent phases of a project aiming to implement interventions, as it enables the tailoring of interventions to context-specific determinants [24], guides choices regarding implementation strategies [25], helps explain implementation outcomes [26] and helps to guide the selection of sustainability strategies [27].

Assessing context is best done by making use of theory driven approaches, as it ensures all relevant aspects of context are studied and enables theoretical development based on empirical evidence [23, 27]. The Consolidated Framework for Implementation Research (CFIR) is one of the most widely used determinant frameworks in implementation science [26, 28, 29]. It offers a list of constructs that promote consistent taxonomy, terminology, and definitions on which a knowledge base of findings across multiple contexts can be built [28].

Despite the relevance of context, it is still often not reported or only moderately described [27, 30, 31]. This study describes the first step in a project aiming to implement evidence based medication adherence interventions in living labs that can be considered early adopters of medication adherence interventions in the Dutch primary care system. This study aims to describe context-specific characteristics in four living labs prior to the implementation of evidence based interventions aiming to improve medication adherence.

## Methods

### Study design

This study used a qualitative design based on semi-structured interviews and focus groups with healthcare providers. The COREQ checklist for qualitative studies was used and can be found in Additional file 1: Appendix A [32].

### Defining context

Context in this study is defined as *‘the environment or setting in which the proposed change is to be implemented.’* [33]. This study operationalizes context by making use of the CFIR [28]. More specifically, this study uses the domains ‘inner setting’ and ‘outer setting’ to define context, omitting the domains ‘intervention characteristics’, ‘characteristics of individuals’ and ‘process’ in the analyses, in line with our chosen definition and following the example of earlier studies using the CFIR to assess context [34–36]. The unit of analysis in this study is the healthcare setting in which the implementation occurs, i.e. the four living labs.

### The four living labs

A research setting that can facilitate a focus on real world aspects is a living lab [37]. Living labs are defined by the European Network of Living Labs as “user centred open innovation ecosystems based on a systematic user co-creation approach, integrating research and innovation processes in real-life communities and settings” (openlivinglabs.eu/aboutus).

The four living labs in this study all applied to and received a grant by ZonMw, the Netherlands Organization for Health Research and Development. As such, they are considered early adopters of medication adherence interventions in the Dutch primary care system. These living labs consisted of cooperating pharmacists and General Practitioners. The goal of these living labs was to implement evidence based medication adherence interventions in the Dutch primary care setting. These were located in four urban areas in the Netherlands and referred throughout the paper as living labs A, B, C and D. Table 1 gives an overview of the four living labs and their characteristics. They were to be guided by the Medication Adherence Knowledge and Expertise and Implementation Taskforce (Make-It consortium). Make-It aims to guide the living labs according to the implementation science methods by Nilsen (2015): 1) guiding and supporting the living labs in translation of research into clinical practice, 2) understand determinants of implementation by examining the contexts of each living labs, and 3) evaluate the actual implementation of interventions within the living labs [38]. All projects ran from December 2020 up until December 2022, with the exception of living lab D which finished in July 2022. Background information about the settings and context of of the four living labs can be found in Additional file 2: Appendix B.

**Table 1:** Characteristics of the four living labs implementing evidence based medication adherence interventions in the Dutch primary care system

	**Living lab A**	**Living lab B**	**Living lab C**	**Living lab D**
**Intervention(s) to be implemented**	TeLCIP	TMI, MeMo and TRIAGE	TelCIP and MeMo	Teach-back method and comprehensible prescription labels
**Project Leader(s)**	Outpatient pharmacist	Community pharmacist	A Community Pharmacist and a policy adviser	A (non-practicing) pharmacist and a community pharmacist
**Involved healthcare providers**	Outpatient pharmacy, 14 community pharmacies and linked GPs	Three community pharmacies and linked GPs	Five community pharmacies and linked GPs, a hospital and a home care organization	Two community pharmacies and their affiliated GPs
**Patient population**	Cardiovascular disease patients at first dispensing of medication	All chronic disease patients using the repeat dispensing service	Cardiovascular disease patients	All patients at first dispensing of medication, of which two third have low health literacy
**Period**	December 2020 up until December 2022	December 2020 up until December 2022	December 2020 up until December 2022	December 2020 up until July 2022

#### Living lab A

Living lab A consisted of an outpatient pharmacy located in a hospital and 14 community pharmacies and linked general practitioners, all governed by the same care organization. In this living lab, a telephone counseling intervention (TelCIP [10]) was implemented by community pharmacists to increase medication adherence of cardiovascular disease patients in transmural care. The patients were prescribed medication in a hospital or outpatient setting and received their first dispense of medication by the outpatient pharmacy, but were treated in a primary care setting by community pharmacists.

#### Living lab B

Living lab B consisted of three community pharmacies and its affiliated general practitioners. All chronic medication users that made use of the pharmacy refill service, in which their prescriptions are automatically renewed and distributed by community pharmacies after a given time period, were invited for a yearly consult. During that consult, pharmacy technicians discussed those patients medication use and improved their medication adherence based on consult preparation (TMI and MeMo) and a practical question set to identify problems (TRIAGE) [11, 12, 39].

#### Living lab C

Living lab C consisted of five community pharmacies and their linked general practitioners that are affiliated with a healthcare partnership organization. A hospital and home care organizations had a supporting role and might be consulted about specific patients. Community pharmacists selected non-adherent cardiovascular disease patients based on pharmacy refill data and used a proactive pharmaceutical care intervention program (MeMo [11]) and a telephone counseling intervention (TelCIP [10]) to give patient-tailored advice for identified barriers to improve medication adherence in a multidisciplinary primary care setting.

#### Living lab D

Two community pharmacies and their affiliated general practitioners, all located in the same neighborhood in which about two thirds of inhabitants have low health literacy, were part of living lab D. The teach-back method and comprehensible prescription labels were implemented to improve patients’ medication adherence by general practitioners and community pharmacy staff members [13, 14]. The teach-back method is a communication technique that involves asking patients to explain medical information in their own words to assess their understanding and promote effective patient-provider communication, known to enhance patients’ health literacy and self-management [40]. Comprehensible prescription labels refer to medication labels that are designed with clearer and understandable language, formatting, and visual aids to enhance patient comprehension and promote safe and effective medication use. This part of the intervention was based on a previous study by one of the project leaders of the living lab [14].

### Recruitment and data collection

**Fig. 1:**
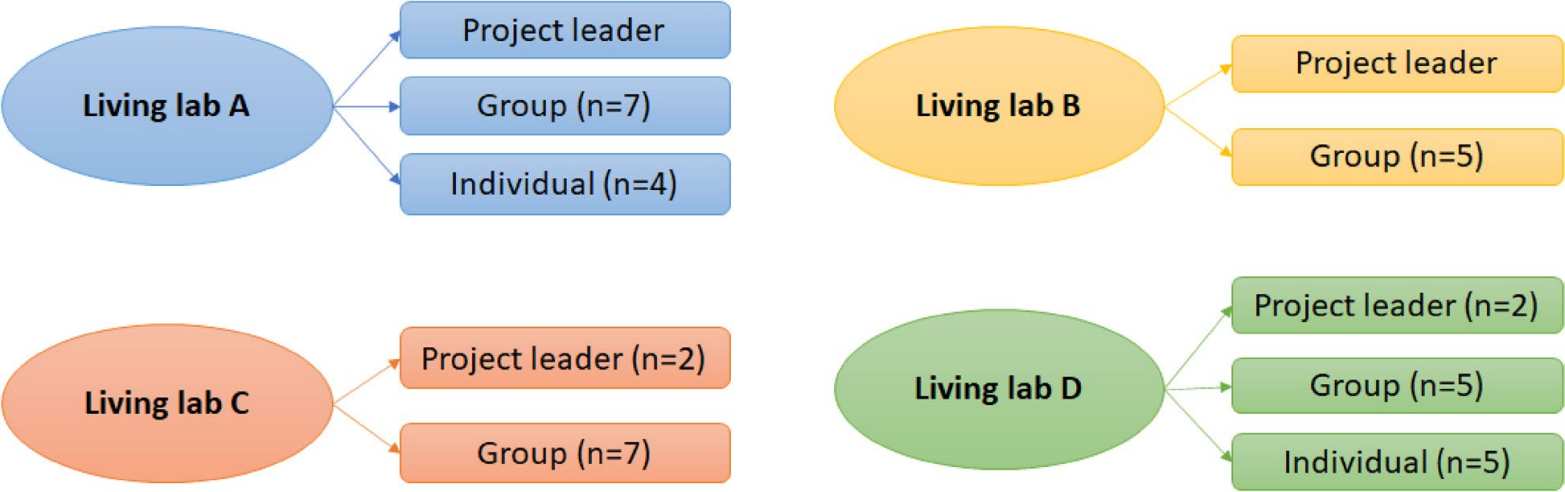
Overview of interviews per living lab

Semi-structured interviews and focus groups were held by members of the Make-It consortium (SH, MV, RJ, CvdS, CB, HZ, LvD). The only in- and exclusion criteria were that respondents were healthcare providers working for the living lab. A total of sixteen interviews were held with project leaders and involved healthcare providers (*N* = 39, community pharmacists, pharmacy technicians, general practitioners and a home care employee) from the four settings. See Fig. 1 for an overview of interviews per living lab. Healthcare providers were selected purposively by the project leaders based on characteristics chosen by the Make-It consortium (i.e. type of employee, experience, attitude towards the project) in order to obtain a diverse group. Focus group interviews (*n* = 4) lasted on average an hour and 13 min. Project leader and individual interviews lasted on average 43 min. Most interviews were held and recorded by video-call (Zoom or Teams), with the exception of two individual interviews that were held at a community pharmacy and audio recorded using voice recorders. All interviews were transcribed verbatim and informed consent was obtained at the start of each interview.

**Fig. 2:**
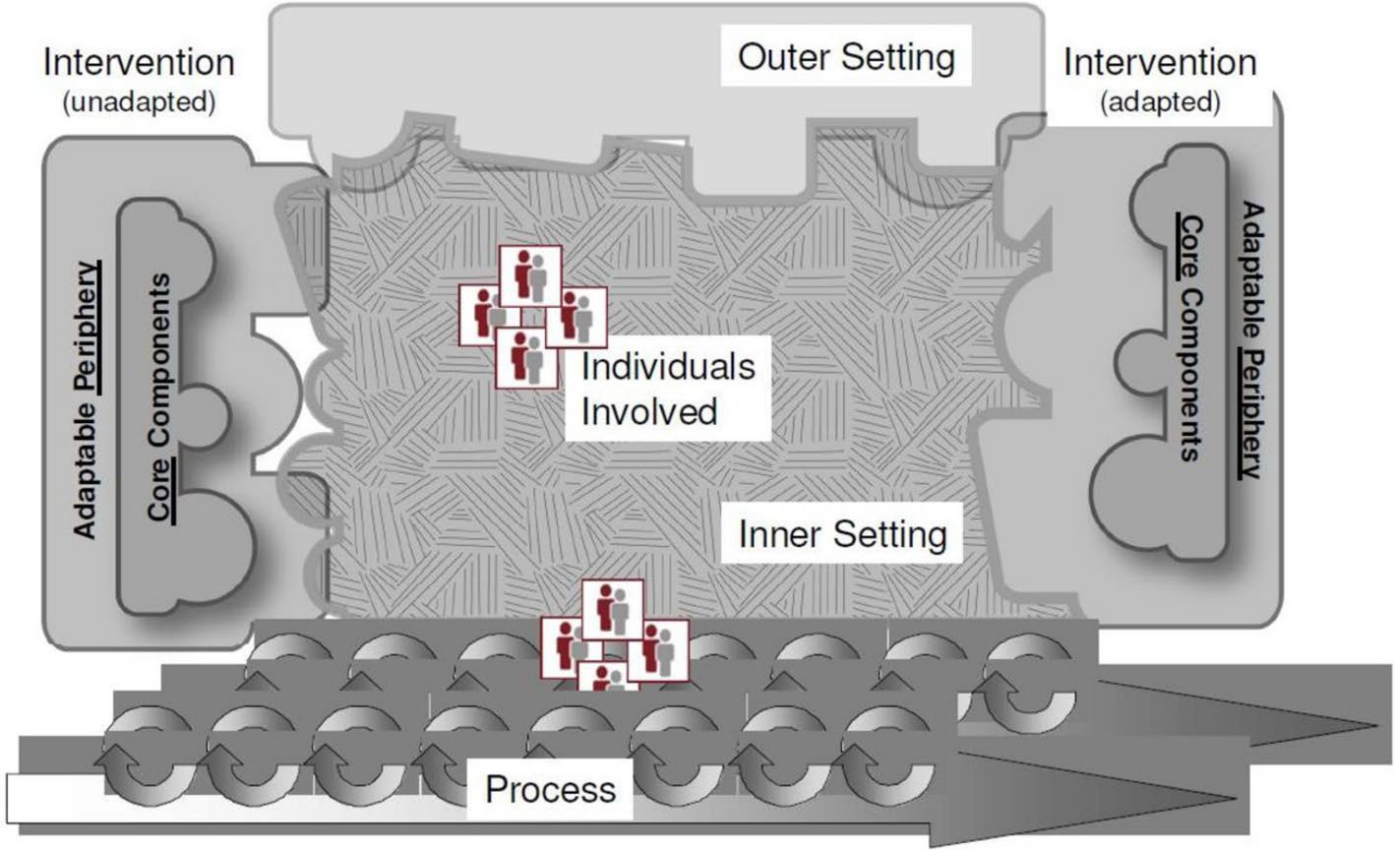
Consolidated framework for Implementation Research (CFIR)

Topic lists were made specific to the type of interview (i.e. project leader, individual and focus group interviews) based on the CFIR and can be found in Additional file 3: Appendix C. Constructs of the CFIR used in the topic lists were chosen by SH, MV and LvD based on relevance for the current study. Chosen constructs could differ between type of interview. This was first done independently after which the chosen constructs were compared between the researchers. Any disagreement was solved through consensus. In the end, the chosen constructs indeed differed between the types of interviews. For example, the CFIR construct ‘leadership engagement’ was asked in individual interviews and focus groups, but not in project leader interviews, whereas ‘external policy andand incentives’ were discussed with project leaders but not with individual healthcare providers. All constructs under the domains ‘inner’ and ‘outer setting’ were included in at least one of the three types of interviews, except the construct ‘peer pressure’. This construct was omitted as the four living labs were selected by the funding body because they are early adopters and therefore peer pressure was not applicable. Choices in topic lists were also made with practical considerations in mind, as in a real world setting healthcare providers’ time is limited and the duration of the interview had to be kept to a minimum. The CFIR guide (cfirguide.org, see Fig. 2 and Additional file 3: Appendix C, [28]) was used to obtain the questions used in the different interviews per construct, which were translated into Dutch by the researchers (SH, MV and LvD).

### Data analyses

All interviews were recorded and transcribed verbatim and analyzed anonymously. Summaries of the records were made by the interviewer, which were member checked by e-mail with the project leaders of the four living labs. Transcripts were analyzed with deductive thematic analysis using the Framework Method [41] and a codebook based on the ‘inner setting’ and ‘outer setting’ domains of the CFIR. SH and MV independently coded the first 3 interviews and then discussed contradictory coding to ensure the different CFIR domains and constructs were coded consistently. Any disagreement was solved through consensus. The remaining interviews were coded by SH and any doubt in coding was discussed with MV and LvD. The CFIR codebook, a template with clear definitions and in- and exclusion criteria of the different CFIR constructs, was used as a reference document during coding of all transcripts. Atlas.ti version 22 was used for the management and coding of transcripts (ATLAS.ti Scientific Software Development, Berlin). Results are organized according to CFIR domains (inner setting andand outer setting) and all of the constructs of these domains [28]. Quotations are used to illustrate the themes and are presented in italics.

## Results

### Participants

A total of 39 people participated in the (group) interviews (Table 2). In living lab A, a project leader interview (*n* = 1), focus group (*n* = 8) and four individual interviews were held. In living lab B we held one project leader interview (*n* = 1) and one focus group (*n* = 5). In living lab C, a project leader interview (*n* = 2) and one focus group (*n* = 7) were held. In living lab D, one project leader interview (*n* = 2), one focus group (*n* = 5) and five individual interviews were held. The interviews lasted 47 min on average with a range from 23 min up to 82 min. The majority of participants were pharmacists (*n* = 12) or pharmacy technicians (*n* = 11). Each living lab had either one or two project leaders, and each living lab had at least one project leader who was also a practicing pharmacist.

**Table 2:** Characteristics of participants of interviews and focus groups per living lab (*N* = 39)

		**Living lab A** (*n* = 12)	**Living lab B** (*n* = 6)	**Living lab C** (*n* = 9)	**Living Lab D** (*n* = 12)
**Gender**	*Male*	1	1	1	1
	*Female*	11	5	8	11
**Function**	*Pharmacist*	8	1	2	2
	*Pharmacy technician*	2	2	2	5
	*GP*	0	1	1	2
	*Practice Nurse*	1	1	1	1
	*Nurse*	0	0	1	0
	*Project leader*^*a*^	1	1	2	2

^a^Project leaders are listed in this table as such, but in 5 out of 6 cases were also pharmacists

The results are structured according to the two CFIR domains inner and outer settings, with the CFIR constructs as sub-themes.

### Outer setting

#### Patient needs and resources

All healthcare providers indicated that the interventions used in their settings addressed an important need of patients. Medication (non-)adherence is seen as a major issue especially by pharmacy staff members who indicate that this can be problematic for patients; they see this problem on a daily basis.

Pharmacist: ‘W*e sometimes think that patients are doing fine, but then suddenly they have built up a medication stock for three or four months, so things are clearly going wrong.’*

Additionally, healthcare providers indicated that patients appreciated the extra care that they received as a result of the intervention.

#### Cosmopolitanism

A large variety in the degree to which healthcare providers networked with external organizations was observed. Project leaders were involved in networks and collaborations more often than the other healthcare providers to discuss the implementation of the interventions with external parties, for example on social media. Other healthcare providers in living labs A and D showed generally lower levels of cosmopolitanism, contrary to living labs B and C. In living lab C specifically, the pharmacy chain to which many community pharmacies belong stimulated that participating healthcare providers posted about the project on social media.

Pharmacy technician: ‘*We are of course stimulated by [Dutch pharmacy chain] to post on Facebook and LinkedIn. You have to be visible as a pharmacy, to show what you can do. I am a big supporter of that.’*

#### External policy and incentives

Multiple pharmacists indicated that financing structures and policy impacted the relative priority of different tasks in the pharmacy, as a pay-for-performance activities had higher priority than other activities, even when these are perceived as equally or even more useful for patients. This latter was also true for the different adherence interventions in the living labs, and multiple healthcare providers expressed their worries that the adherence intervention(s) might not continue without any external financing structures.

Pharmacist: *‘….It’s all very much focused on earning money with the things that we do. That applies to this* [i.e. the project] *as well. Of course we received a grant, but we did not arrange any financing structures for when the grant ends, and we’re already discussing whether we should continue to do this’.*

### Inner setting

#### Structural characteristics

Living lab D consisted of two multicultural pharmacies, both in its staff and its patients. In other living labs, participating pharmacies differ in their population, dependent on the neighborhood in which the pharmacy is located. Living lab A was recently reorganized into clusters of pharmacies, each managed by a single community pharmacist. These clusters are characterized by less autonomy for individual community pharmacies within these clusters, but more centralized governance and improved collaboration between community pharmacies in the same cluster.

#### Networks and communications

Communication between pharmacy technicians from different pharmacies is rare. Community pharmacists and general practitioners communicated with each other through larger meetings, such as pharmacy audit meetings in which pharmacotherapeutic decisions are made and the projects were discussed. Specific peer meetings were set up for participating pharmacists to reflect on the project’s progress in the two larger living labs A and C. The smaller living labs B and D were characterized by more informal communication. For example, they used WhatsApp, phone calls or face-to-face contact.

Pharmacist: *‘…On Mondays I have my lunch walk with [project leader], where we can discuss things about the project.’*

#### Culture

The way in which healthcare providers perceived the overall working climate was positive, they indicate a lot of respect for their coworkers and a pleasant working environment.

Pharmacy technician: ‘*There is respect here amongst each other for sure. We let each other finish sentences. The general atmosphere is very positive and some people have worked here for 25 or even 40 years, that I think says something’.*

On top of that, multiple healthcare providers from living lab A mentioned a more rigid management approach, primarily aimed at improving the financial situation of the organization. As stated earlier, they have recently had a change in governance, where a single pharmacist is now in charge of a cluster of multiple community pharmacies. Healthcare providers mentioned that these changes, as well as some other choices for innovations such as a medicine locker, a locker in which patients can pick up medication themselves 24 h a day, were primarily financially driven.

Pharmacist: ‘*It is all about money. About earning money with all the different things that we do. Within this organization, if there is no financing, it will not continue. That is the current policy.‘*

#### Implementation climate

All living labs showed a high regard for innovation, and indicated that the role of the pharmacist should be shifting towards more patient oriented care.

Pharmacist: *‘…If I look back at the past few years, we keep improving. You would almost forget it, but we have introduced a medicine locker since two months, and that project is going well. We have started working paperless before that, so you simply go along with all sorts of improvements. It all happens very naturally, we are never standing still‘.*

Despite that each living lab was a multidisciplinary project, the living labs were predominantly community pharmacist-driven. general practitioners interviewed in this project all indicated that they perceive the projects as useful and addressing important problems in patients, but also indicated that the most important role is for the pharmacy staff members. In all four projects, pharmacy staff members performed consultations with patients which can traditionally be seen as a role for general practitioners, but the mutual trust and level of collaboration between general practitioners and community pharmacy staff members within the four living labs was such that general practitioners did not mind that shift.

general practitioner: *‘I think that* [i.e. not being involved in the meeting] *would be best, because we do not really need to know how the project is organized and how and which patients need to be invited and that sort of stuff. I think it is fine if we receive an update or a phone call about difficult patients of ours from the pharmacy every now and again.’*

##### Tension for change

Healthcare providers indicate that they see non-adherence daily in their practice, and that evidence based interventions are available but are not being used. They clearly see areas in which care can and should be improved, such as more patient support at first and second dispensing of medication or for patients going from secondary to primary care.

Pharmacist: *‘…and the thought that there is something* [i.e. an intervention] *available on the shelf that is not being applied in daily practice. That is a thought that motivated me to join this project. On top of that, a patients transferal from a medical specialist in the hospital setting to the patients’ home setting and a community pharmacy is known to be the cause of many issues, and this project can help to improve that transfer for the patient’.*

##### Compatibility

All four living labs had past experience in projects aimed at medication adherence and improving patient oriented care, such as implementing medication adherence interventions in their settings previously or adding other health innovations such as medicine lockers to their pharmacies. The topics that are addressed by the different medication adherence interventions are in line with the needs of the living labs. They were therefore highly compatible according to the involved healthcare providers.

Pharmacist: *‘The teach-back method I think is very suitable for our location, because we are all interested in low health literacy and how we can improve our communication. In fact, we have already done this for years, and I think you can see this project as an extension of what we have been doing all along.’*

Additionally, living labs B and C aimed to increase the compatibility of the intervention with the work flow in individual community pharmacies by allowing freedom in the way the intervention was performed. Individual pharmacies could for example choose their own target population or type of employee responsible for doing the consultations.

##### Relative priority

As stated before, living lab A saw a shift in organizational structure where a single community pharmacist is now managed by a single community pharmacist, which many healthcare providers perceived to be a possible risk in priority.

Pharmacist: ‘*There are no other current projects that require a lot of time, but the discipline of pharmacy in our care group has seen a large reorganization of the management structure, which is an important risk factor for this project.’*

The other living labs had either no other main priorities or did not perceive them to be a threat to implementation of the interventions.

##### Organizational incentives and rewards

Tangible incentives such as promotions or salary increases for individual employees were not applied as rewards for carrying out the interventions, and performance reviews in general were rare for pharmacy staff members. However, there were some implicit signals that involvement in the project lead to increased status or respect, as some pharmacy technicians had dedicated roles in promoting the innovation.

Pharmacy technician: *‘And I of course do not really know yet what it all means and how we are going to do it, but I am appointed as quality technician and my role is to get the other technicians excited for this project. And I think that he* [the project leader]* sees that this sometimes works and sometimes doesn’t, but usually it does work, and that is why he asked me’.*

##### Goals and feedback

The importance of setting goals by the project leaders was highlighted by multiple healthcare providers in the project.

Pharmacist: *‘I think a risk for this project could be that the management simply lets the project organize itself and is not on top of it. If that might happen, I can already see that a week goes by very quickly in a community pharmacy where the aim is to work on the project, but when it is very busy at the front desk, the technicians might simply not carry out the intervention.’*

These goals were set by the different project leaders in the living labs, as they facilitated (online) platforms in which healthcare providers could keep track of goals. These platforms were used to give feedback to the involved healthcare providers. Different living labs had different methods of keeping track of goals and giving feedback. Examples are keeping track of project outcomes with planning software and giving feedback based on these results (living lab A), making one pharmacist responsible for reaching project goals in their cluster of pharmacies (living lab B), regular video call meetings in which outcomes per pharmacy practice were discussed (living lab C) or discussing project goals in daily morning meetings at the pharmacy (living lab D).

##### Learning climate

All living labs fostered a learning climate, for example by emphasizing that all healthcare providers play an important part and that it is okay to make mistakes. Most examples of a learning climate come from living labs B and D. In living lab D, pharmacy technicians feel their views on the project were valued by community pharmacists. In living lab B, the project leader was deliberately trying to foster a learning climate by delegating responsibilities to different healthcare providers.

Pharmacist: *‘This is a RASCI-table, which stands for Responsible, Accountable, Consultable and Informed. So that is something I use to divide the tasks in this project. The goal is to involve everyone in the project, and to make agreements clear from the start. So I want to make someone responsible per result, and have at least one person be supportive to him or her.’*

#### Readiness for implementation

The project leaders made efforts to involve (higher) management and different healthcare providers in the commitment to carry out the interventions. This led to clarity for all healthcare providers in the living labs, as everyone was participating in the projects. This was visible when pharmacy technicians showed a ‘just do it’ attitude:

Pharmacy technician*: ‘It started simply on the first day. We were informed about the project during work meeting, and immediately started applying it* [the teach-back method]* at the front desk the next day.’*

##### Leadership engagement

All project leaders were seen by healthcare providers as being easy to approach. For the two larger living labs A and C, not everyone had a direct working relationship with the project leaders, but in those cases healthcare providers could address their questions to another leadership figure such as a their own community pharmacist. In living lab D, pharmacy technicians noticed a high level of involvement of the community pharmacist.

Pharmacy technician: *‘For example with the prescription, we did not know yet that we had to indicate on each prescription that we conducted the teach-back method and how long it took us. That was immediately addressed by the community pharmacist, and from there on out we were strictly checked on this.’*

The project leaders of living labs B and D offered a large degree of freedom for individual community pharmacies to tailor the intervention to the specific context of the different practices.

Pharmacist: ‘*I think it is good to find your own way per community pharmacy, that’s fine. I think that* [project leader] *has deliberately not put us into boxes, as I don’t think that’s necessary’.*

In living lab A, a threat to good leadership engagement was a community pharmacist who did not have a positive attitude towards the project. This was especially threatening because this pharmacist was also a manager of one of the clusters of participating community pharmacies.

##### Available resources

All living labs have received funding by Dutch research funder ZonMw for carrying out the projects, which allowed community pharmacists to dedicate staff to the projects. Despite that, there were worries both in living lab C and D that some involved community pharmacies experienced a shortage of staff, or had a high turnover of staff which caused new pharmacy technicians not to be equipped to carry out the interventions.

Pharmacist: *‘You need to have enough time available for technicians to carry out the intervention. And that technician also needs to have followed the training. So you need to have sufficient time for that in your pharmacy. So especially now in the summer period, where there is a high turnover of staff, I think this could be a barrier.’*

##### Access to knowledge and information

By the time of the (group) interviews, participants in living labs A and D had already participated in a training, whereas living lab B and C had a training planned which had not yet taken place. These trainings were held by external trainers, and healthcare providers were generally positive about the training they received.

After the initial training, the living labs had different methods of providing information about how to implement the interventions in daily practice. In the two larger living labs A and C, a core project group organized information about the intervention using formal methods, such as through online guides or news letters to share updates on the project. The two smaller living labs B and D had more informal ways to communicate about how to carry out the intervention.

Pharmacist: ‘*In SAS* [name of business analytics software]. *All information can be found there, and you can filter per pharmacy. So the phone number, type of medication, the patient, it’s all in there. So when* [name of pharmacist] *has to call patients, she can use this to make a selection’.*

## Discussion

This study aimed to describe context-specific characteristics in four living labs prior to the implementation of evidence based medication adherence enhancing interventions. The results show that all living labs were pharmacy driven and can be characterized by a high regard for innovation by staff members, a positive implementation climate, high levels of leadership engagement and high compatibility between the living labs and the chosen interventions. Worries about external policy, most notably lack of reimbursement for sustainment and upscaling of the interventions, were shared among all living labs. The fact that the four living labs contexts share several favorable characteristics fits their position as early adopters when it comes to the application of medication adherence interventions. Rogers describes early adopters as people who need little incentive to try out new innovations [42]. In his diffusion of innovations theory, adoptability of innovations is further facilitated through a perceived tension for change and innovation-system fit (compatibility) on a system level and a high regard for the innovation by individual healthcare providers, which all apply to these living labs. Despite the theoretical guidance on characteristics of early adopting settings, little is known about which of these characteristics are actually linked to implementation success and what the underlying mechanisms are [26]. Future studies should therefore aim to associate CFIR constructs with project outcomes, in order to learn what contextual factors should be altered to positively influence implementation [28]. However, authors have noted that cultivating a practice culture that values innovations is difficult [43], and focusing on early adopters first and subsequently describing a pathway for less adopting practices to tag along with might be more beneficial [44]. Such a pathway would require describing the context of less adopting settings in detail as well and pinpointing which aspects of their context needs improving in order to facilitate successful implementation. This is a next step in our study, as four new, less experienced living labs will start their projects in the winter 2022–2023.

Although the current living labs shared characteristics that are favorable for good implementation, a few notable differences in the context of the four living labs were observed as well. Living labs A and C were the two larger living labs in terms of number of involved community pharmacies and other practices and in the way they were organized. These larger living labs showed more formal ways of working in project planning, networks and communications and goal setting, while the two smaller living labs B and D used more informal communication methods. Besides the scale, living labs B and C were found to be more cosmopolitan than living labs A and D. Additionally, project leaders from living labs B and C offered a larger degree of freedom for individual community pharmacies in their living lab to tailor the intervention to their context. This adaptability is an important aspect of both implementation and further upscaling and dissemination in the future [28, 45], especially for more complex interventions, such as the telephone counseling intervention in which multiple healthcare providers from different practices have to collaborate [10].

All four living labs were pharmacy-driven (i.e. pharmacists as project leaders) with a supporting role for general practitioners and other primary care healthcare providers. Patient consults in which medication adherence is discussed are more traditionally held by general practitioners, but in the specific context of these four living labs, general practitioners had no reservations to delegate some of these consults to pharmacy staff members. This was rooted in high trust and a strong collaboration that had built up during past projects between pharmacy and general practitioner staff members in all four living labs. This shift in more patient-oriented care for pharmacists is a trend seen worldwide that is also encouraged by the Dutch umbrella organization for pharmacists [46, 47]. However, in their systematic review, Weir et al. have shown that a common barrier for implementing innovations in the community pharmacy setting is limited engagement of patients and general practitioners in the community pharmacy [48]. Our study has given a positive exception to this finding by showing that early adopting pharmacies can play an important role in patient-oriented care. The established involvement of both patients and general practitioners in the community pharmacy setting might be an aspect of the Dutch primary care setting, as previous innovations such as the nationwide implementation of the clinical medication review has provided community pharmacies with this experience [49].

A concern shared by healthcare providers of all four living labs was external policy, especially the lack of structural funding and the emphasis on financial goals in the pharmacy setting. The sustainment of health interventions often relies on external funding, and is a common concern in multiple implementation projects [30, 31, 50]. Involving important stakeholders such as health insurers and policy makers from an early stage onwards is recommended [30], but a recent study showed that the web of stakeholders all involved in pharmaceutical care is highly complicated [51].

### Strengths and limitations

Using the CFIR as theoretical guidance both in data collection and analyses increased the consistency of results, enabled a greater understanding of implementation characteristics and promoted the rigor and trustworthiness of the research [23, 26, 52, 53]. Additionally, a detailed definition of context and a clear description of the unit of analysis served as theoretical guidance throughout the study [23, 33].

Some limitations in the data collection should be noted. The project leader interviews and focus groups were held before healthcare providers had experience with the interventions, whereas the individual interviews were held after the healthcare providers had already started with the implementation. However, by focusing solely on the context by means of the CFIR domains ‘inner’ and ‘outer settings’ and not on other CFIR domains such as ‘process’, the different phases of the project and performance of the interventions are not the focus of this study. Additionally, due to limited availability of healthcare providers, individual interviews with respondents other than project leaders were only performed in the living labs A and D and not in the two others. This led to more data on two of the four living labs. However, sufficient data was available for all living labs to describe them in detail using the CFIR. Transcripts were send back to project leaders for member checking. This may have impacted the responses of other participants, or have harmed their privacy. However, transcripts were anonymized before sending back to project leaders.

Additionally, the four living labs provide examples of positive implementation settings, as all of these four living labs are considered early adopters, and results cannot be generalized to all other settings for implementation of medication adherence enhancing interventions, specifically to less experienced settings.

Lastly, all four living lab projects were performed in times of covid lockdowns, which is the reason that most interviews were performed by means of video calls. While live interviews are considered superior for obtaining information from participants, differences between them are marginal [54].

## Conclusions

The context for the implementation of medication adherence enhancing intervention in four living labs that were considered early adopters in the field when receiving an implementation grant is favorable, even though they differed in levels of scale and cosmopolitanism. The results of this study provide detailed examples of a positive implementation setting. These can be used to inform dissemination of medication adherence interventions in settings less experienced in implementing medication adherence interventions. Future studies should assess the feasibility to perform these interventions in more average performing pharmacy settings, as well as link the context-specific characteristics provided in this study to project outcomes, in order to assess the influence different contextual determinants have on implementation.

### Supplementary Information

**Additional file 1. **COREQ (COnsolidated criteria for REporting Qualitative research) Checklist.

**Additional file 2: Appendix B.** Background information for the four living labs.

**Additional file 3: Appendix C. **CFIR domains used as topic lists in project leader, individual and group interviews to assess context of four living labs implementing pharmaceutical interventions in primary care [28].

## Data Availability

In order to safeguard the privacy of our participants, data and transcripts are not made available.

## Abbreviations

CFIR: Consolidated Framework for Implementation Research
RCT: Randomized controlled trial
