# Supplementary material for: Implementing medication adherence interventions in four Dutch living labs; context matters
Source: BMC Health Serv Res. 2023 Sep 26;23:1030. doi: 10.1186/s12913-023-10018-4 (PMC10523767; doi:10.1186/s12913-023-10018-4)
Supplement: Supplementary file 2 — Additional file 2: Appendix B. Background information for the four living labs. [file 12913_2023_10018_MOESM2_ESM.docx]

Appendix B background information for the four living labs.

**Living lab A**

This living lab is situated in a medium sized city and is governed by an umbrella organization. Rather than living lab C, this umbrella organization is focused solely on healthcare. All pharmacies in the city are participants, which means they collaborate in different combinations depending on their locations within the city.

**Living labs B and D**

The two small living labs (B and D) are neighborhood-based, and the pharmacists know each other and collaborate within the neighborhood. The contexts in which they operate, however, differ: one is in a low wealth neighborhood, while the other is not, the demography mainly consists of young families. One is in a large city, while the other is in a medium-sized city. Furthermore, all pharmacies operate independently and can determine staffing and other aspects on their own. They also have this autonomy within the living lab.

**Living lab C**

Living lab C operates from an overarching collaboration of all health organizations in a big city, governed by an umbrella organization that also includes home care practices. There are five participating pharmacies, but they don't collaborate in day-to-day practice due to being located in different parts of the city. There has been an effort to ensure variation across neighborhoods by deliberately including 5 varying community pharmacies. The pharmacies here also have the autonomy to determine their own approach.
