# Supplementary material for: Implementing medication adherence interventions in four Dutch living labs; context matters
Source: BMC Health Serv Res. 2023 Sep 26;23:1030. doi: 10.1186/s12913-023-10018-4 (PMC10523767; doi:10.1186/s12913-023-10018-4)
Supplement: Supplementary file 3 — Additional file 3: Appendix C. CFIR domains used as topic lists in project leader, individual and group interviews to assess context of four living labs implementing pharmaceutical interventions in primary care [28]. [file 12913_2023_10018_MOESM3_ESM.docx]

**Appendix C.** CFIR domains used as topic lists in project leader, individual and group interviews to assess context of four living labs implementing pharmaceutical interventions in primary care [28].

| **Consolidated Framework for Implementation Research Constructs** |
| --- |
|  |
| [CFIR Website](http://cfirguide.org) |

| **Construct** | | **Short Description** | **Type of interview the construct was applied in** |
| --- | --- | --- | --- |
| **I. INTERVENTION CHARACTERISTICS** | |  |  |
| A | Intervention Source | Perception of key stakeholders about whether the intervention is externally or internally developed. | Not included in interviews |
| B | Evidence Strength & Quality | Stakeholders’ perceptions of the quality and validity of evidence supporting the belief that the intervention will have desired outcomes. | Not included in interviews |
| C | Relative Advantage | Stakeholders’ perception of the advantage of implementing the intervention versus an alternative solution. | **Included in project leader and group interviews** |
| D | Adaptability | The degree to which an intervention can be adapted, tailored, refined, or reinvented to meet local needs. | **Included in group interviews** |
| E | Trialability | The ability to test the intervention on a small scale in the organization, and to be able to reverse course (undo implementation) if warranted. | Not included in interviews |
| F | Complexity | Perceived difficulty of implementation, reflected by duration, scope, radicalness, disruptiveness, centrality, and intricacy and number of steps required to implement. | **Included in group interview** |
| G | Design Quality & Packaging | Perceived excellence in how the intervention is bundled, presented, and assembled. | Not included in interviews |
| H | Cost | Costs of the intervention and costs associated with implementing the intervention including investment, supply, and opportunity costs. | Not included in interviews |
| **II. OUTER SETTING** | |  |  |
| A | Patient Needs & Resources | The extent to which patient needs, as well as barriers and facilitators to meet those needs, are accurately known and prioritized by the organization. | **Included in group interviews** |
| B | Cosmopolitanism | The degree to which an organization is networked with other external organizations. | **Included in group interviews** |
| C | Peer Pressure | Mimetic or competitive pressure to implement an intervention; typically because most or other key peer or competing organizations have already implemented or are in a bid for a competitive edge. | Not included in interviews |
| D | External Policy & Incentives | A broad construct that includes external strategies to spread interventions, including policy and regulations (governmental or other central entity), external mandates, recommendations and guidelines, pay-for-performance, collaboratives, and public or benchmark reporting. | **Included in project leader interviews** |
| **III. INNER SETTING** | |  |  |
| A | Structural Characteristics | The social architecture, age, maturity, and size of an organization. | **Included in group interviews** |
| B | Networks & Communications | The nature and quality of webs of social networks and the nature and quality of formal and informal communications within an organization. | **Included in individual interviews** |
| C | Culture | Norms, values, and basic assumptions of a given organization. | **Included in individual interviews** |
| D | Implementation Climate | The absorptive capacity for change, shared receptivity of involved individuals to an intervention, and the extent to which use of that intervention will be rewarded, supported, and expected within their organization. | **Included in individual interviews** |
| 1 | Tension for Change | The degree to which stakeholders perceive the current situation as intolerable or needing change. | **Included in group interviews** |
| 2 | Compatibility | The degree of tangible fit between meaning and values attached to the intervention by involved individuals, how those align with individuals’ own norms, values, and perceived risks and needs, and how the intervention fits with existing workflows and systems. | **Included in group interviews** |
| 3 | Relative Priority | Individuals’ shared perception of the importance of the implementation within the organization. | **Included in individual and group interviews** |
| 4 | Organizational Incentives & Rewards | Extrinsic incentives such as goal-sharing awards, performance reviews, promotions, and raises in salary, and less tangible incentives such as increased stature or respect. | **Included in individual and group interviews** |
| 5 | Goals and Feedback | The degree to which goals are clearly communicated, acted upon, and fed back to staff, and alignment of that feedback with goals. | **Included in group interviews** |
| 6 | Learning Climate | A climate in which: a) leaders express their own fallibility and need for team members’ assistance and input; b) team members feel that they are essential, valued, and knowledgeable partners in the change process; c) individuals feel psychologically safe to try new methods; and d) there is sufficient time and space for reflective thinking and evaluation. | **Included in individual interviews** |
| E | Readiness for Implementation | Tangible and immediate indicators of organizational commitment to its decision to implement an intervention. | **Included in project leader and group interviews** |
| 1 | Leadership Engagement | Commitment, involvement, and accountability of leaders and managers with the implementation. | **Included in individual and group interviews** |
| 2 | Available Resources | The level of resources dedicated for implementation and on-going operations, including money, training, education, physical space, and time. | **Included in group interviews** |
| 3 | Access to Knowledge & Information | Ease of access to digestible information and knowledge about the intervention and how to incorporate it into work tasks. | **Included in group interviews** |
| **IV. CHARACTERISTICS OF INDIVIDUALS** | |  |  |
| A | Knowledge & Beliefs about the Intervention | Individuals’ attitudes toward and value placed on the intervention as well as familiarity with facts, truths, and principles related to the intervention. | **Included in individual interviews** |
| B | Self-efficacy | Individual belief in their own capabilities to execute courses of action to achieve implementation goals. | **Included in individual interviews** |
| C | Individual Stage of Change | Characterization of the phase an individual is in, as he or she progresses toward skilled, enthusiastic, and sustained use of the intervention. | Not included in interviews |
| D | Individual Identification with Organization | A broad construct related to how individuals perceive the organization, and their relationship and degree of commitment with that organization. | **Included in individual interviews** |
| E | Other Personal Attributes | A broad construct to include other personal traits such as tolerance of ambiguity, intellectual ability, motivation, values, competence, capacity, and learning style. | Not included in interviews |
| **V. PROCESS** | |  |  |
| A | Planning | The degree to which a scheme or method of behavior and tasks for implementing an intervention are developed in advance, and the quality of those schemes or methods. | Not included in interviews |
| B | Engaging | Attracting and involving appropriate individuals in the implementation and use of the intervention through a combined strategy of social marketing, education, role modeling, training, and other similar activities. | Not included in interviews |
| 1 | Opinion Leaders | Individuals in an organization who have formal or informal influence on the attitudes and beliefs of their colleagues with respect to implementing the intervention. | **Included in group interviews** |
| 2 | Formally Appointed Internal Implementation Leaders | Individuals from within the organization who have been formally appointed with responsibility for implementing an intervention as coordinator, project manager, team leader, or other similar role. | **Included in group interviews** |
| 3 | Champions | “Individuals who dedicate themselves to supporting, marketing, and ‘driving through’ an [implementation]” [101] (p. 182), overcoming indifference or resistance that the intervention may provoke in an organization. | **Included in group interviews** |
| 4 | External Change Agents | Individuals who are affiliated with an outside entity who formally influence or facilitate intervention decisions in a desirable direction. | **Included in group interviews** |
| C | Executing | Carrying out or accomplishing the implementation according to plan. | Not included in interviews |
| D | Reflecting & Evaluating | Quantitative and qualitative feedback about the progress and quality of implementation accompanied with regular personal and team debriefing about progress and experience. | Not included in interviews |
